# Pathological and Inflammatory Consequences of Aging

**DOI:** 10.3390/biom15030404

**Published:** 2025-03-12

**Authors:** Mario García-Domínguez

**Affiliations:** 1 Program of Immunology and Immunotherapy, CIMA-Universidad de Navarra, 31008 Pamplona, Spain; mgdom@unav.es; 2 Department of Immunology and Immunotherapy, Clínica Universidad de Navarra, 31008 Pamplona, Spain; 3 Centro de Investigación Biomédica en Red de Cáncer (CIBERONC), 28029 Madrid, Spain

**Keywords:** inflammation, inflammaging, aging, proteostasis, telomere shortening, cellular senescence, immunosenescence, adipaging, parthanatos

## Abstract

Aging is a complex, progressive, and irreversible biological process that entails numerous structural and functional changes in the organism. These changes affect all bodily systems, reducing their ability to respond and adapt to the environment. Chronic inflammation is one of the key factors driving the development of age-related diseases, ultimately causing a substantial decline in the functional abilities of older individuals. This persistent inflammatory state (commonly known as “inflammaging”) is characterized by elevated levels of pro-inflammatory cytokines, an increase in oxidative stress, and a perturbation of immune homeostasis. Several factors, including cellular senescence, contribute to this inflammatory milieu, thereby amplifying conditions such as cardiovascular disease, neurodegeneration, and metabolic disorders. Exploring the mechanisms of chronic inflammation in aging is essential for developing targeted interventions aimed at promoting healthy aging. This review explains the strong connection between aging and chronic inflammation, highlighting potential therapeutic approaches like pharmacological treatments, dietary strategies, and lifestyle changes.

## 1. Introduction

The process of aging constitutes a multifaceted and intricate phenomenon characterized by a gradual deterioration of several biological functions [1,2], thereby rendering humans more susceptible to a range of age-associated diseases [3]. This mechanism involves many molecular, cellular, and systemic alterations that progressively impair overall functionality [4,5,6]. At the molecular level, aging is linked to genomic instability [7], resulting in the accumulation of DNA damage [8]. Consequently, the DNA damage leads to telomere shortening [9] and epigenetic alterations that regulate gene expression [10]. On the other hand, aging is linked to strong reduction in proteostasis, which impairs cells’ ability to maintain a functional proteome [11]. Additionally, mitochondrial dysfunction occurs, leading to a decrease in energy production and an increased generation of reactive oxygen species (ROS) that damage cellular structures [12].

These changes result in cellular senescence, a state in which cells cease to divide but remain metabolically active [13]. Senescent cells exhibit a senescence-associated secretory phenotype (SASP), characterized by the secretion of pro-inflammatory cytokines (such as IL-1α, IL-1β, IL-6, IL-8, and IL-18), growth factors (e.g., VEGF, TGF-β, GM-CSF, IGFBP-2, and GDF-15), and proteases [14,15,16]. The accumulation of senescent cells within tissues is now widely recognized as a key driver of aging and the development of many conditions like cardiovascular diseases, neurodegeneration, and cancer [17,18,19]. The sustained secretion of the mentioned SASP factors occupies an essential position in the formation and continuation of a chronic inflammatory milieu; this environment contributes to both peripheral and central inflammatory mechanisms [20].

At the peripheral level, the release of SASP mediators significantly amplifies systemic inflammation, triggering a cascade of physiopathological events that disrupt normal tissue homeostasis [21]. However, this persistent inflammatory state causes structural and functional alterations in tissues, driving pathological remodeling in organs such as the heart, liver, and kidneys. Pro-inflammatory mediators promote fibrosis and ischemic injury by inducing maladaptive repair processes while simultaneously impairing the regenerative processes necessary for maintaining tissue integrity. As a result, this situation creates a cycle of dysfunction that accelerates tissue damage and hinders proper recovery [22,23]. This biological process, known as inflammaging, is characterized by chronic low-grade inflammation associated with aging, sustained by the SASP mediators [24,25]. This condition not only perpetuates tissue damage but also amplifies the degenerative changes associated with aging, establishing a vicious cycle of inflammation and dysfunction that impedes tissue repair and accelerates the progression of age-related diseases.

At the central nervous system (CNS) level, SASP mediators can exert significant effects on the brain. One of the major consequences is the disruption of the blood–brain barrier (BBB), a critical defense mechanism that protects the brain from harmful substances present in the bloodstream [26]. The prolonged inflammation caused by SASP mediators can compromise the integrity of the BBB; thus, it facilitates the infiltration of peripheral immune cells, toxins, and pro-inflammatory cytokines into the CNS [27]. This dysregulation within the CNS not only initiates neuroinflammation but also strongly activates microglial cells. In their activated state, these cells release additional pro-inflammatory cytokines, further exacerbating neurodegenerative processes linked to aging such as Parkinson’s and Alzheimer’s diseases [28,29]. Microglial activation induces several effects, like synaptic dysfunction, neuronal cell death, and disruptions in neuronal signaling [30,31]. This aberrant inflammatory response may accelerate neuronal loss, impair cognitive function, and evoke the accumulation of toxic protein aggregates, such as β-amyloid plaques in Alzheimer’s disease and α-synuclein in Parkinson’s disease [32,33].

This review will provide a comprehensive analysis of the pathophysiological consequences of aging, emphasizing the molecular, cellular, and systemic alterations that drive age-related deterioration. Furthermore, this review will investigate the inflammatory processes linked to aging, both at the peripheral and central levels. Particular emphasis will be placed on the underlying mechanisms that drive inflammation, including immune cell activation, oxidative stress, and the accumulation of senescent cells. By synthesizing current research findings, this review aims to provide a deeper understanding of the interplay between aging and inflammation, shedding light on potential therapeutic interventions to mitigate the adverse effects of these processes.

## 2. Aging: Physiopathological Consequences

Aging is a process that leads to functional physiological changes, including increased susceptibility to many chronic conditions [3]. The complex physiopathological processes linked to aging influence multiple body systems, such as the cardiovascular, nervous, immune, and musculoskeletal systems, thereby elevating the risk of neurodegeneration, cardiovascular diseases, and metabolic disorders, among others [34]. Gaining a deep understanding of the physiological consequences of aging is essential for developing effective therapeutic approaches that not only support healthy aging but also mitigate the impact of age-related diseases. As the population ages, it becomes increasingly important to identify and address the underlying biological mechanisms that contribute to the decline in organ function, immune system efficiency, and cognitive abilities. By prioritizing these processes, researchers can design interventions that promote longevity, enhance quality of life, and reduce the burden of chronic diseases, ultimately helping older individuals maintain their independence and well-being as they age [35].

### 2.1. Nervous System

The human body undergoes numerous alterations within its CNS throughout the aging process, leading to changes in cognitive functions, memory capabilities, and overall brain health status [36]. A key factor in this process is the increase in oxidative stress. On a molecular level, oxidative stress is critical, as aging neurons accumulate ROS due to mitochondrial dysfunction [37]. The brain experiences elevated levels of malondialdehyde (MDA) along with lipid peroxidation products such as 4-hydroxy-2-nonenal (4-HNE), which induce DNA damage and promote lipid peroxidation and protein misfolding factors involved in neurodegenerative processes [38,39].

**Figure 1:**
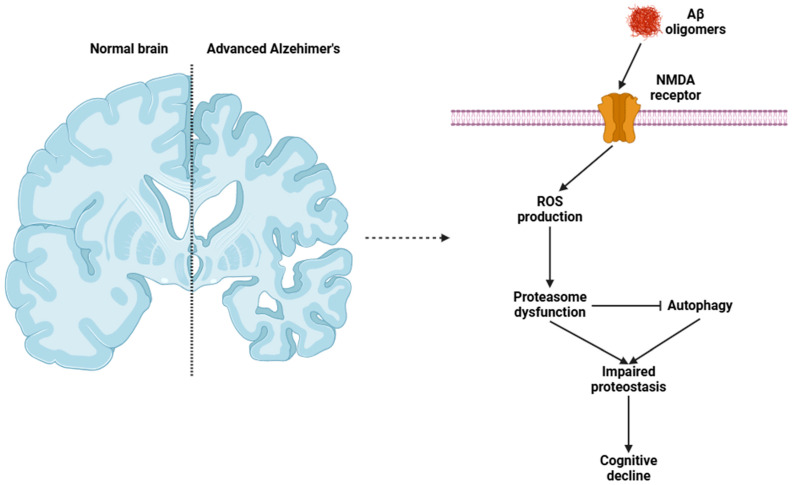
Cellular mechanisms that occur in the CNS neurons of individuals with Alzheimer’s disease. Abbreviations: NMDA (N-methyl-D-aspartate) and ROS (reactive oxygen species).

Telomere shortening is a fundamental marker of aging that amplifies neuronal vulnerability by restricting the regenerative potential of neural stem cells and reducing synaptic plasticity while also inhibiting neurogenesis in the hippocampus, a crucial brain region for learning and memory [40,41]. Conversely, the balance of proteostasis, involving protein synthesis, folding, and degradation, is disrupted during aging as the functional efficiency of both the ubiquitin–proteasome system and autophagy declines [42]. This deficiency facilitates the accumulation of protein aggregates such as β-amyloid plaques seen in Alzheimer’s disease (Figure 1) and α-synuclein aggregates found in Parkinson’s disease [32,33].

Furthermore, reduced levels of neurotransmitters such as dopamine, acetylcholine, and serotonin can disrupt signal transmission, resulting in several problems with motor coordination, mood regulation, and cognitive function [43]. The myelin sheath is likewise affected by dysfunctions in oligodendrocytes, resulting in delayed neural conduction and increased susceptibility to demyelinating diseases [44]. Neuroinflammation, primarily driven by activated microglia and astrocytes, accelerates neural aging through the release of various pro-inflammatory cytokines such as IL-6, IL-1β, and TNF-α [45]. These cytokines can impair synaptic function and contribute to neuronal degeneration [46]. Furthermore, aging influences several signaling pathways that are fundamental for normal neuronal function, including the PI3K-AKT and MAPK pathways [47]. Additionally, the age-related decline in BBB integrity aggravates neurodegenerative conditions by facilitating the infiltration of peripheral immune cells and toxins into the central nervous system, triggering chronic inflammation and neuronal injury [27].

On the other hand, the buildup of advanced glycation end-products (AGEs) also negatively impacts neuronal function by causing oxidative stress and persistent inflammation [48]. Finally, the meninges, the protective layers surrounding the brain and spinal cord, undergo age-related changes in gene expression that may modify the brain’s microenvironment and contribute to age-related neurological decline [49].

### 2.2. Cardiovascular System

The aging process profoundly affects the cardiovascular system through a complex interplay of molecular and cellular mechanisms, resulting in structural and functional alterations that compromise both cardiac and vascular integrity [50]. At the molecular level, collagen accumulation and impaired cardiomyocyte function play a critical role in the development of left ventricular hypertrophy and myocardial fibrosis [51]. Additionally, mitochondrial dysfunction, which involves decreased ATP production and the increased generation of ROS, disrupts cellular energy balance and worsens oxidative stress [52]. The blood vessels also experience significant transformations, such as reduced nitric oxide availability due to lower activity of endothelial nitric oxide synthase (eNOS) and increased nitric oxide depletion from ROS [53]. Chronic low-grade inflammation, driven by the SASP phenotype, along with impaired angiogenesis resulting from diminished vascular endothelial growth factor (VEGF) signaling, further exacerbates cardiovascular dysfunction [54].

Telomere shortening in vascular cells accelerates cellular senescence and increases the risk of apoptosis [55]. Epigenetic alterations, including changes in DNA methylation, histone modifications, and dysregulated microRNA expression, modulate gene activity linked to oxidative stress, fibrosis, and inflammation [56]. The accumulation of AGEs promotes the crosslinking of extracellular matrix proteins, leading to increased arterial stiffness and diastolic dysfunction [57]. Additionally, impaired protein maintenance and autophagy lead to the accumulation of misfolded proteins and damaged organelles, accelerating cellular dysfunction and disease progression [58].

### 2.3. Gastrointestinal System

The aging process has a significant effect on the gastrointestinal (GI) system, leading to functional decline and a higher risk of diseases through various molecular mechanisms [59]. One major factor is the degeneration of neuromuscular function, which includes the loss of interstitial cells of Cajal and a reduction in acetylcholine signaling, resulting in impaired peristalsis and contributing to constipation [60,61]. Esophageal issues, particularly the weakening of the lower esophageal sphincter, are influenced by changes in nitric oxide signaling, which raises the risk of gastroesophageal reflux disease (GERD) [62]. Furthermore, gastric acid secretion decreases due to atrophic gastritis, which is frequently linked to inflammation caused by *Helicobacter pylori* and an increase in pro-inflammatory cytokines like IL-1β, IL-2, IL-4, IL-6, IL-8, IL-10, IFN-γ, and TNF-α [63]. The reduction in acid secretion can interfere with nutrient absorption [64].

In the pancreas, mitochondrial dysfunction and endoplasmic reticulum stress lead to a significant reduction in enzyme secretion from the pancreatic islets, resulting in the malabsorption of macronutrients [65,66]. The liver also suffers from decreased blood flow and reduced activity of cytochrome P450 enzymes, especially CYP3A4, which affects drug metabolism and raises the risk of medication toxicity [67]. On the other hand, the colon undergoes structural weakening due to strong alterations in extracellular matrix remodeling, driven by matrix metalloproteinases, thereby increasing the risk of diverticulosis and inflammatory complications [68]. The risk of colorectal cancer also rises due to accumulated DNA damage, telomere shortening, and many mutations in tumor suppressor genes such as *TP53*, worsened by the chronic activation of NF-κB and the overproduction of prostaglandin E2 [69,70]. Moreover, polypharmacy can disrupt gut health, as NSAIDs inhibit COX-1 and raise the risk of ulcers, while antibiotics can lead to dysbiosis, increasing susceptibility to *Clostridioides difficile* infections [71,72]. Dehydration, resulting from reduced sensitivity of hypothalamic osmoreceptors, exacerbates colonic transit, leading to more severe constipation and increased systemic inflammation [73].

Ultimately, the gut–brain axis plays a critical role in the aging process, impacting cognitive function, mental health, and overall well-being [74]. The gut microbiome, a complex community of trillions of microorganisms residing in the digestive tract, promotes bidirectional communication with the brain via the gut–brain axis, an interconnected network that includes the vagus nerve, immune system, and neuroactive compounds such as neurotransmitters and short-chain fatty acids (SCFAs) [75]. With age, the gut microbiome undergoes profound changes, often exhibiting decreased diversity due to factors such as an imbalanced diet, medication use (particularly antibiotics), and decreased physical activity [76]. Alterations in the gut microbiota, characterized by a strong reduction in *Bifidobacteria* and *Lactobacillus* populations, activate Toll-like receptor (TLR) pathways and compromise gut barrier integrity by downregulating tight junction proteins such as occludin and claudin-1 [77,78,79]. This situation contributes to systemic inflammation, immune dysregulation, and cognitive decline [80]. Additionally, the gut microbiota regulates the production of some neurotransmitters, including serotonin and dopamine, which participate in mood regulation and cognitive function [81]. Finally, aging is associated with a reduction in gut barrier integrity, resulting in increased intestinal permeability, which enables harmful substances to enter the bloodstream and trigger immune responses that could accelerate neurodegeneration [82].

### 2.4. Respiratory System

As individuals age, the respiratory system undergoes several physiological changes that progressively reduce pulmonary function, increasing susceptibility to respiratory illnesses and impairing oxygenation efficiency [83,84]. The primary age-related alteration is the gradual loss of lung elasticity, driven by structural modifications in elastin and collagen fibers within lung tissue [85], due to the deposition of collagen and the degradation of elastin fibers. This change is caused by an imbalance between matrix metalloproteinases (MMPs), enzymes responsible for degrading extracellular matrix (ECM) components, and their tissue inhibitors (TIMPs) [86], which disrupt the structural integrity of the lungs, leading to decreased lung compliance and a reduction in alveolar surface area [87].

In the alveolar region, aging is associated with a reduction in the production of surfactants (such as dipalmitoylphosphatidylcholine—DPPC) and several surfactant proteins [88]. This leads to an altered composition of the surfactant, resulting in a less efficient reduction in surface tension in the alveolar sacs. Consequently, this alteration in surfactant composition not only compromises alveolar integrity but also hinders effective gas exchange by reducing the surface area [89].

The chest wall becomes increasingly rigid due to costal cartilage calcification and structural changes in the intercostal muscles, limiting thoracic expansion and decreasing the capacity for deep inhalation [90]. The diaphragm may also experience functional decline due to age-related sarcopenia, further weakening inspiratory and expiratory forces [91]. Moreover, aging negatively impacts pulmonary immune defense mechanisms, including a significant reduction in mucociliary clearance, thereby increasing vulnerability to respiratory infections such as pneumonia and chronic obstructive pulmonary disease (COPD) [92]. Finally, the sensitivity of central and peripheral chemoreceptors responsible for detecting fluctuations in blood oxygen and carbon dioxide levels diminishes with age, leading to a blunted ventilatory response to hypoxia and hypercapnia [93].

### 2.5. Urogenital System

In the urinary tract, age-related changes manifest as a decrease in renal mass, particularly in the renal cortex, leading to a reduction in the number of functioning nephrons [94,95]. This decline in the nephron population contributes to a gradual decrease in the glomerular filtration rate and renal blood flow, impairing the kidneys’ ability to concentrate urine, regulate fluid balance, and maintain electrolyte homeostasis [96]. As a result, older adults may experience diminished renal clearance of waste products, making them more susceptible to dehydration, drug accumulation, and electrolyte imbalances, which can further exacerbate comorbid conditions such as hypertension and diabetes [97,98]. Additionally, the dysregulation of the renin–angiotensin–aldosterone system (RAAS) and endothelial dysfunction, marked by reduced nitric oxide production, further compromise renal function in older individuals [99,100].

The bladder also undergoes significant structural and functional changes with aging. A decrease in detrusor muscle mass, along with increased collagen deposition in the bladder wall, leads to reduced bladder compliance and impaired contractility [101]. These alterations, combined with numerous age-related neurological changes affecting autonomic bladder control, contribute to lower bladder capacity and an increased prevalence of urinary symptoms like frequency, urgency, nocturia, and incomplete emptying [102]. Additionally, age-related weakening of the urethral sphincter and pelvic floor muscles in men and women increases the risk of urinary incontinence, which can impact the overall quality of life and psychological well-being [103,104].

In men, the prostate gland commonly enlarges with age due to benign prostatic hyperplasia (BPH), which may produce urinary obstruction, hesitancy, a weak stream, and post-void dribbling [105]. In BPH, the overexpression of HSP70 subfamily members contributes to cell survival, proliferation, and epithelial–mesenchymal transition, promoting prostate enlargement [106]. Oxidative stress plays a fundamental role, with a considerable accumulation of ROS resulting in mitochondrial dysfunction and, subsequently, cellular damage [107]. Additionally, the activation of the CXCL12/CXCR4 axis moderates prostate myofibroblast phenoconversion through non-canonical EGFR/MEK/ERK signaling, contributing to tissue fibrosis [108]. If BPH is left untreated, severe cases lead to chronic urinary retention and an increased risk of urinary tract infections, bladder stones, and kidney damage [109,110,111]. In contrast, the urogenital system in older women undergoes numerous changes, particularly post menopause, due to reducing estrogen levels [112]. This hormonal shift significantly contributes to the atrophy of the vaginal and urethral epithelia, leading to dyspareunia, vaginal dryness, increased susceptibility to bacterial and fungal infections, and decreased urethral resistance, thereby increasing the risk of stress and incontinence [113]. The loss of estrogen also accelerates connective tissue degeneration, increasing the risk of pelvic organ prolapse [114].

The impact of aging extends to the reproductive system, where a progressive decline in fertility is observed in both sexes [115]. In men, testicular function diminishes, resulting in reduced testosterone production, a lower sperm count, and a decrease in sperm motility, which can contribute to subfertility [116]. Although older men retain reproductive potential throughout life, these changes may lead to decreased sexual function, including reduced libido and an increased prevalence of erectile dysfunction, often influenced by vascular, neurological, and several metabolic factors [117,118]. Women experience a more abrupt transition with menopause, marked by the cessation of ovarian function and reproductive capability [119]. The depletion of ovarian follicles leads to a substantial decline in estrogen and progesterone levels, which may reduce antioxidant capacity, thereby increasing oxidative damage. This process promotes a pro-inflammatory state characterized by elevated cytokine production, including IL-6 and TNF-α, with systemic effects on bone density and cardiovascular function [119,120,121].

### 2.6. Endocrine System

The aging process profoundly affects the endocrine system, resulting in significant physiological and biochemical changes that disrupt homeostasis, metabolism, and overall health [122]. At a molecular level, aging results in the dysregulation of hormonal signaling pathways, often manifested by diminished hormone production, reduced receptor sensitivity, and impaired feedback systems [123]. A critical factor in this decline is the gradual deterioration of hypothalamic function, which leads to a decrease in the secretion of regulatory hormones such as gonadotropin-releasing hormone (GnRH), growth hormone-releasing hormone (GHRH), and corticotropin-releasing hormone (CRH) [124]. This reduction leads to lower levels of peripheral hormones, including growth hormone (GH), insulin-like growth factor 1 (IGF-1), and sex steroid hormones (androgens, estrogens, and progestogens) [125].

Several factors, such as cellular senescence, oxidative stress, and inflammaging, further worsen endocrine dysfunction by impairing the responsiveness of endocrine glands [126]. Furthermore, aging induces certain post-translational modifications (PTMs), leading to alterations in proteins that affect cellular signaling, enzymatic activity, and hormone receptor functionality, further contributing to endocrine decline [127]. These PTMs include glycation, a non-enzymatic process in which reducing sugars, such as glucose and fructose, covalently bind to proteins, lipids, or nucleic acids [128]. This process results in the formation of advanced glycation end-products (AGEs), a diverse group of molecular complexes that progressively accumulate with age [129]. AGEs play a key role in cellular dysfunction, particularly in the endocrine system, as they can modify hormone receptors, disrupt their normal functioning, and alter the structure of circulating hormones [130].

The aging of pancreatic β-cells is associated with mitochondrial impairment, endoplasmic reticulum stress, and epigenetic changes, which collectively diminish insulin secretion and heighten the risk of developing type 2 diabetes [131]. In the adrenal glands, age-related modifications include disrupted cortisol secretion due to altered sensitivity to adrenocorticotropic hormone (ACTH), exacerbating stress responses and metabolic irregularities [132]. Similarly, the reduction in dehydroepiandrosterone (DHEA) secretion negatively impacts immune function and tissue repair [133]. On the other hand, changes in thyroid function, particularly the reduced bioconversion of thyroxine (T4) to triiodothyronine (T3), lead to metabolic slowdowns and greater vulnerability to age-related illnesses [134].

### 2.7. Musculoskeletal System

The aging process evokes a cascade of structural and functional changes that significantly reduce mobility, strength, and overall quality of life [135]. These modifications are driven by a complex interplay of mechanisms that together contribute to the progressive decline in musculoskeletal health. Among the most prominent of these factors is oxidative stress. Oxidative damage affects mitochondria, decreasing ATP production and impairing muscle function while reducing cellular energy. Moreover, the decline in repair mechanisms with age worsens the buildup of damaged molecules, organelles, and tissues [136,137].

In skeletal muscle, one of the hallmark consequences of aging is sarcopenia, characterized by the progressive loss of muscle mass and function [138]. Sarcopenia is driven by several factors, including a decrease in the number and size of muscle fibers, particularly fast-twitch type II fibers, which are responsible for rapid, high-intensity muscle contractions. This decline in muscle fibers results in reduced strength and endurance, making daily activities more difficult [139]. The ability of muscle tissue to repair itself also diminishes with age, due in part to a decline in satellite cell function [140]. Satellite cells play a critical role in muscle regeneration and repair; however, with advancing age, their ability to proliferate and differentiate into new muscle fibers decreases substantially [141]. Aging also leads to an imbalance between muscle protein synthesis and protein degradation. The reduced biosynthesis of muscle proteins and the increased breakdown of existing muscle proteins contribute to muscle atrophy [142]. Moreover, age-related hormonal changes, including a reduction in GH and testosterone levels, further impair muscle anabolism and regeneration, intensifying the overall loss of muscle mass and function [143].

Simultaneously, aging has significant consequences for bone health. In bone tissue, aging leads to an imbalance between bone formation and resorption, favoring bone loss and increasing the risk of fractures [144]. Osteoblasts, the cells responsible for bone formation, decrease in number and activity with age, while osteoclasts, the cells responsible for breaking down bone, either remain unchanged or increase in activity [145]. Oxidative stress is a key factor in this process, where increased levels of ROS cause cellular damage and induce apoptosis in osteocytes [146]. Simultaneously, there is a reduction in the osteogenic capacity of bone marrow stromal cells, indicated by a reduced expression of Runx2, an essential regulator of osteoblast differentiation [147]. The alteration in bone metabolism is also influenced by age-related changes in systemic hormones, such as estrogen, testosterone, and growth hormone, as well as local growth factors that regulate bone turnover [148,149]. Another contributor to age-related bone fragility is the accumulation of AGEs in collagen fibers within the bone matrix, leading to the increased rigidity and decreased elasticity of collagen. This process causes the bone matrix to become more brittle and more prone to fractures, further weakening bone strength [150].

The ECM also undergoes several changes with age in both muscle and bone [151,152]. In muscle tissue, the ECM becomes increasingly fibrotic, which reduces its elasticity and impairs its ability to support muscle regeneration and contraction [153]. Muscle fibrosis limits the capacity of muscle tissue to contract efficiently, contributing to reduced strength and function [154]. Additionally, changes in the composition and function of MMPs contribute to the remodeling of muscle tissue and the degradation of some structural proteins [155]. Similarly, in bone tissue, aging leads to diverse alterations in collagen crosslinking and biomineralization patterns, influencing the mechanical properties of bone [156]. These changes in the bone ECM are driven by shifts in the expression and activity of MMPs, further contributing to the increased fragility of bones in older individuals [157].

In muscle and bone tissues, epigenetic changes can disrupt the production of essential proteins necessary for tissue regeneration and maintenance, accelerating the decline in tissue function [158]. The accumulation of senescent cells in muscle and bone tissues releases several pro-inflammatory cytokines and MMPs, which aggravate tissue degeneration and impair the repair capacity of the musculoskeletal system [159].

Finally, as individuals age, the connection between the brain and muscles becomes progressively crucial for maintaining overall physical health and functional independence [160]. The brain regulates muscle movement through a network of neurons that transmit electrical signals from the CNS to activate muscle fibers and coordinate voluntary movements. However, with aging, this communication system may become less efficient [161]. The motor cortex, which is responsible for planning and executing movement, may experience a reduction in both the quantity and effectiveness of neural connections, leading to slower reaction times, impaired coordination, and a decreased ability to perform complex motor tasks [162]. The decline in neural and muscular functions elevates the risk of falls, diminishes mobility, and adversely impacts quality of life [163].

## 3. Chronic Inflammation in Aging

The relationship between aging and peripheral inflammation represents a complex and multifactorial process, with many molecular mechanisms contributing to a prolonged state of chronic low-grade inflammation, also called inflammaging [24,25,164,165,166]. In contrast to acute inflammation, which is a transient response to infection or injury [167], inflammaging is a persistent, low-grade inflammatory state that develops as a result of the combined influence of internal and external factors accumulated throughout life. This process is characterized by sustained immune pathway activation, the increased production of pro-inflammatory cytokines, and the dysregulation of immune homeostasis, all of which contribute to the progressive functional decline associated with aging [168].

Aging affects multiple peripheral organs, including the liver, adipose tissue, skeletal muscles, and gastrointestinal tract, all of which play a crucial role in modulating systemic inflammation [17,18,19]. The progressive dysfunction of these organs with age is primarily caused by molecular and cellular alterations, including oxidative stress, genomic instability, epigenetic changes, mitochondrial impairment, and cellular senescence [1,2,169]. All of these create an inflammatory microenvironment that evokes tissue damage, ultimately contributing to the onset and progression of many age-related diseases, including cardiovascular disorders, neurodegenerative conditions, and cancer [170,171,172]. At the molecular level, inflammaging involves a complex network of inflammatory mediators, including cytokines, acute-phase proteins, and DAMPs, which activate various intracellular signaling pathways [7,8,9,10,11,12,173].

A defining feature of inflammaging is the SASP phenotype. As aging occurs, senescent cells accumulate in several tissues, promoting a pro-inflammatory environment that supports immune system activation and drives tissue remodeling [13,14,15,16,174]. An additional factor in inflammaging is gut microbiota dysbiosis, which has become increasingly recognized as a significant regulator of systemic inflammation in aging individuals [175]. Age-related alterations in gut microbiota composition can result in increased intestinal permeability, facilitating the translocation of bacterial endotoxins, such as lipopolysaccharide (LPS), into the circulation [176]. This process, called metabolic endotoxemia, triggers sustained immune cell activation, further enhancing systemic inflammation [177].

Nevertheless, chronic inflammation in the CNS of older adults is primarily mediated by the accumulation of SASP mediators, which can cross the BBB and activate glial cells, thereby contributing to the detrimental effects of the inflammatory process. These factors not only exacerbate the neuroinflammatory response but also create a cycle of sustained immune activation that further impairs brain health, making it a significant factor in age-related neurological disorders [26,27].

### 3.1. Peripheral Inflammation

**Figure 2:**
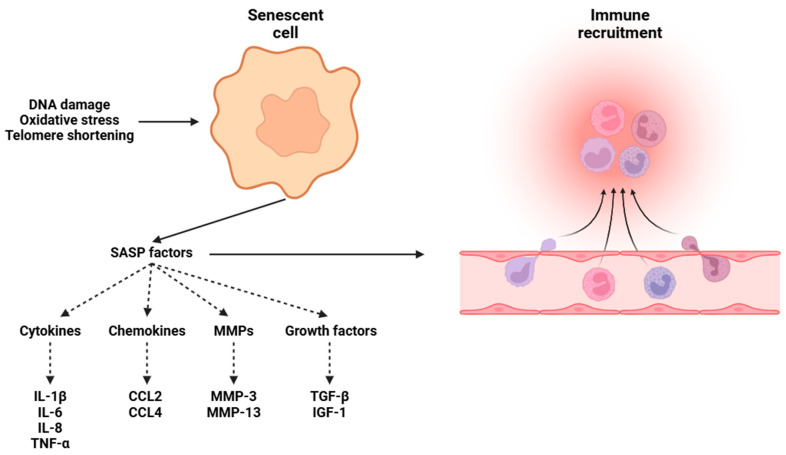
The process through which senescent cells secrete SASP mediators in response to damage caused by oxidative stress, telomere shortening, and DNA impairment. Abbreviations: DNA (deoxyribonucleic acid), IL-1β (Interleukin 1 beta), IL-6 (Interleukin 6), IL-8 (Interleukin 8), TNF-α (tumor necrosis factor alpha), CCL2 (C-C motif chemokine ligand 2), CCL4 (C-C motif chemokine ligand 4), MMP-3 (matrix metalloproteinase 3), MMP-13 (matrix metalloproteinase 13), TGF-β (transforming growth factor beta), and IGF-1 (insulin-like growth factor 1).

Chronic inflammation is considered one of the hallmarks of aging, and a key player in this process is the accumulation of senescent cells [20]. Cellular senescence is a state in which cells cease proliferation, frequently in response to many stressors, including DNA damage, telomere shortening, and oxidative stress [7,8,9,10,11,12]. These cells show high metabolic activity; they lose their ability to replicate, which makes them resistant to apoptotic signals [13]. Senescent cells (Figure 2) adopt an active state, undergoing some changes in gene expression rather than remaining inactive, thereby promoting the senescence-associated secretory phenotype (SASP). Senescent cells secrete several pro-inflammatory cytokines, including IL-6 and TNF-α, both of which play essential roles in inflammation and immune responses [178,179]. In addition to IL-6 and TNF-α, SASP factors encompass other molecules, such as IL-1β and IL-8, chemokines (e.g., CCL2 and CCL4), MMPs (e.g., MMP-3 and MMP-13), and growth factors (e.g., TGF-β and IGF-1), amplifying local inflammation and contributing to tissue remodeling and degradation [178,180,181,182]. As people age, the activity of the ubiquitin–proteasome system (UPS) decreases, leading to the strong accumulation of polyubiquitinated proteins. This decline is associated with structural changes in the proteasome, the reduced expression of proteasome subunits, and oxidative damage to proteasomal components [183]. In parallel, both autophagy and chaperone-mediated autophagy (CMA) show age-related dysfunctions, leading to a decreased formation of autophagic vacuoles and delayed fusion of autophagosomes with lysosomes, worsening the inflammatory process [184]. Furthermore, the accumulation of ROS stimulates the DNA damage response (DDR) pathway, leading to the stabilization of p53 and the upregulation of p16 and p21 [185]. The activation of p16 and p21 leads to a permanent and stable cell cycle arrest, which is a critical characteristic of senescent cells [186]. This growth arrest is mediated through the inhibition of cyclin-dependent kinases, particularly CDK4/6, leading to the inhibition of retinoblastoma protein (Rb) phosphorylation, a vital regulator of the G1/S transition in the cell cycle [187]. Furthermore, the activation of TLRs and the NLRP3 inflammasome by DAMPs released from senescent cells further amplifies the inflammatory response [188].

A key characteristic of SASP mediators is their capacity to evoke autocrine and paracrine signaling, where the signals released by senescent cells affect healthy cells [185,189]. For instance, the pro-inflammatory cytokines secreted by senescent cells can promote the activation of immune cells, such as macrophages and T lymphocytes, which in turn release more pro-inflammatory molecules, exacerbating the inflammatory environment [190,191]. The presence of SASP mediators in some tissues, such as adipose tissue, skeletal muscle, liver, and the vasculature, has been linked to several major age-related diseases [192]. In atherosclerosis, senescent cells located in the blood vessel walls contribute to the chronic inflammation that underlies plaque formation [14]. Furthermore, in osteoarthritis, senescent cells situated in the cartilage secrete factors that degrade the ECM, leading to joint dysfunction and pain [193]. Although senescence serves as a protective function by preventing the proliferation of damaged cells, its presence has detrimental long-term effects. The aging immune system becomes less efficient at clearing senescent cells, which leads to the exacerbation of inflammation [194]. In this context, aging significantly influences the clearance of damaged cells. The reduced effectiveness of immunosurveillance leads to a decreased ability of the body to identify and eliminate damaged cells [195].

A critical molecular pathway that contributes significantly to aging-related inflammation is the involvement of the NF-κB transcription factor, which plays a crucial role in the immune system’s response to stress, injury, and infection [196]. While this response is protective in younger individuals, its persistent activation in the elderly leads to an overzealous immune reaction that contributes to chronic low-grade inflammation [197]. NF-κB, activated by several stimuli (such as pro-inflammatory cytokines and ROS), triggers a cascade of downstream signaling events that enhance the production of pro-inflammatory mediators and adhesion molecules in neutrophils (such as E-selectin and VCAM-1) [198]. Conversely, one of the most consequential adverse effects of prolonged activation is the dysregulation and functional impairment of regulatory CD4^+^ CD25^+^ T cells (Tregs), which are essential for maintaining immune balance by controlling excessive immune responses [199]. With advancing age, Tregs experience a decline in function, reducing their ability to regulate immune activation. This dysfunction in Tregs leads to the breakdown of the finely tuned regulation of immune responses, promoting an imbalance in CD4+ T-cell activity and exacerbating tissue damage [199,200]. Additionally, CD4^+^ CD25^+^ Tregs are susceptible to mitochondrial dysfunction, which is characterized by decreased mitochondrial protein levels and impaired oxidative phosphorylation, along with increased mitochondrial oxidative stress [201]. Tregs undergo alterations in mitochondrial dynamics, including a reduction in mitochondrial mass, a compromised membrane potential, and the accumulation of damaged mitochondria with abnormal morphology, resulting in instability in FoxP3 expression and a reduced capacity to sustain immune homeostasis [202]. Furthermore, mitochondrial dysfunction in aging Tregs is further amplified by impairments in mitophagy (the process responsible for eliminating damaged mitochondria), leading to a self-perpetuating cycle of oxidative damage and cellular senescence [201].

Nevertheless, the aging immune system undergoes additional functional alterations, commonly referred to as immunosenescence. This process encompasses the reduction in the efficiency and capacity of immune responses [203]. In the innate immune system, macrophage and neutrophil activity decreases due to reduced signaling from GM-CSF and impaired TLRs function in monocytes, macrophages, and dendritic cells (DCs). Therefore, these immune cells become less efficient at detecting and responding to pathogens, resulting in a delayed immune response [204]. Likewise, the adaptive immune system suffers a decline in T- and B-cell functionality, compromising their ability to recognize specific pathogens and generate many specific immune responses [205]. This impairment manifests as a reduced ability to produce antibodies after vaccination or infection, making older individuals more susceptible to many infections, including influenza and pneumonia [206].

On the other hand, the molecular mechanisms driving age-related inflammation and vascular dysfunction involve a network of interconnected pathways, each contributing to the multifaceted phenotype of aging. At the epigenetic level, DNA methylation undergoes significant changes with age, marked by global hypomethylation and site-specific hypermethylation [207]. These alterations lead to the activation of numerous pro-inflammatory genes, such as *IL-6*, *TNF-α*, and *COX-2*, while simultaneously suppressing anti-inflammatory factors like IL-10 and TGF-β [208,209]. Histone modifications are vital in chromatin remodeling during aging, with a strong reduction in H3K9 trimethylation and an increase in H4K16 acetylation being particularly notable [210,211,212]. Moreover, the expression of non-coding RNAs, especially microRNAs, is altered with age. For instance, the upregulation of miR-21 promotes vascular inflammation by targeting PTEN and activating the AKT pathway, while the downregulation of miR-126 impairs endothelial function by reducing VEGF signaling [213,214].

The interaction between inflammation and obesity in aging creates a network of molecular processes that significantly impacts health and longevity. Obesity, a metabolic disorder in the elderly [215], induces adipocyte hypertrophy and hyperplasia, leading to a significant increase in the secretion of pro-inflammatory adipokines such as leptin and resistin [216,217]. These adipokines initiate a cascade of inflammatory events, activating mainly mast cells and CD4^+^ T cells to release numerous pro-inflammatory cytokines, including IL-6, IL-12, IL-17, IL-18, and TNF-α [218]. Simultaneously, the expansion of adipose tissue linked to obesity results in localized hypoxia, activating the NF-κB pathway and further enhancing the production of pro-inflammatory mediators [219]. Furthermore, excess adipose tissue acts as an endocrine organ, secreting bioactive molecules that disturb the metabolic balance, including altered lipid metabolism and increased circulating free fatty acids, exacerbating oxidative stress and mitochondrial dysfunction in immune cells [220]. The cumulative effect of these processes establishes a self-perpetuating cycle of inflammation and metabolic dysregulation, known as adipaging, which not only accelerates the aging process but also elevates the risk of age-related diseases such as type 2 diabetes [221].

### 3.2. Central Inflammation

The detrimental influence of SASP mediators on the CNS arises through intricate molecular mechanisms that undermine the structural and functional integrity of the BBB. The BBB is a highly specialized and selective permeability barrier composed of endothelial cells interconnected by tight junctions, primarily consisting of transmembrane proteins such as claudins, occludin, and zonula occludens (ZO) proteins, supported by pericytes and astrocytes [222]. Under physiological conditions, this barrier plays an essential role in maintaining CNS homeostasis by regulating the exchange of ions, nutrients, and signaling molecules while restricting the entry of circulating immune cells and neurotoxic agents [223]. However, chronic exposure of the BBB to SASP factors impairs its selective permeability, resulting in widespread neurovascular dysfunction [224]. As the BBB integrity declines with age, peripheral inflammatory mediators and immune cells are able to infiltrate the CNS [225].

Among the key SASP factors implicated in BBB breakdown are a range of pro-inflammatory cytokines, including IL-1β, IL-6, and TNF-α, in addition to MMPs (such as MMP-2 and MMP-9), which directly degrade tight junction proteins [226]. This disruption facilitates the increased permeability of the BBB, allowing the infiltration of peripheral immune cells, ROS, and additional inflammatory mediators into the CNS parenchyma [227]. The extravasation of these harmful agents exacerbates neuroinflammation by triggering the activation of resident microglial cells, which play a crucial role in the innate immune response within the CNS [228]. Microglial activation is mainly mediated via TLR signaling cascades and the NF-κB transcriptional pathway, leading to a feedforward loop of pro-inflammatory cytokine release that reinforces neuronal damage [229]. The mentioned cytokines activate microglia through TLR4, resulting in the nuclear translocation of NF-κB and subsequent amplification of inflammatory responses [230]. Simultaneously, the activation of the NLRP3 inflammasome in microglia enhances the release of mature IL-1β, perpetuating neuroinflammation [231].

**Figure 3:**
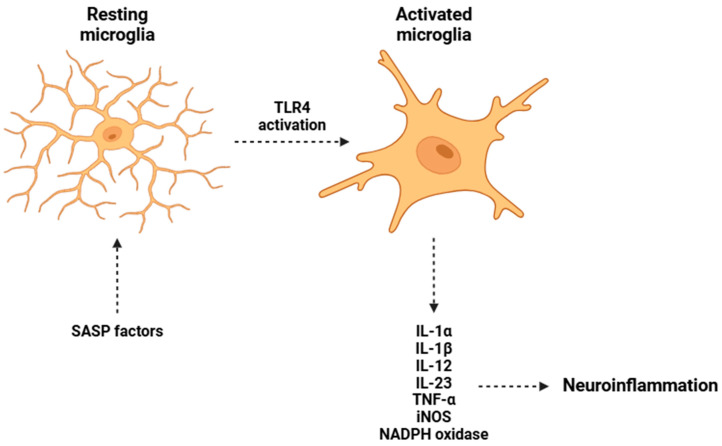
Microglial activation induced by SASP mediators in resting-state microglia. The activation of TLR4 receptors by SASP factors stimulates microglial activation and the release of various pro-inflammatory cytokines. Abbreviations: IL-1α (Interleukin 1 alpha), IL-1β (Interleukin 1 beta), IL-12 (Interleukin 12), IL-23 (Interleukin 23), TNF-α (tumor necrosis factor alpha), iNOS (inducible nitric oxide synthase), and NADPH (nicotinamide adenine dinucleotide phosphate—reduced form).

A consequence of sustained microglial activation (Figure 3) is the excessive production of reactive oxygen and nitrogen species (ROS and RNS), mainly mediated by inducible nitric oxide synthase (iNOS) and NADPH oxidase [232,233]. This overproduction creates a highly pro-inflammatory and cytotoxic environment within the CNS, disrupting cellular homeostasis. The accumulation of ROS exacerbates mitochondrial dysfunction, leading to impaired ATP synthesis and energy deficits in neurons. The energy deficiency compromises several neuronal processes, including synaptic transmission, plasticity, and cell viability [234]. Oxidative stress disrupts several redox-sensitive signaling pathways, triggering molecular damage like DNA oxidation, lipid peroxidation, and protein misfolding [235].

In addition to mitochondrial dysfunction, the interplay between neuroinflammation and oxidative stress exacerbates excitotoxicity. Overactivated microglia evoke excessive glutamate release while simultaneously impairing its reuptake by astrocytes, resulting in glutamate accumulation within the synaptic cleft [236]. This causes the overactivation of NMDA and AMPA receptors, leading to an aberrant influx of calcium into neurons [237]. The increased intracellular calcium activates multiple neurotoxic pathways, including the stimulation of calpains and caspases (proteolytic enzymes that degrade essential cellular components and drive apoptotic and necrotic cell death) [238,239].

At the molecular level, elevated calcium influx disrupts calcium-dependent signaling cascades, exacerbating mitochondrial stress and stimulating the additional release of ROS [240]. This feedback loop perpetuates oxidative damage and cellular dysfunction, creating a self-sustaining cycle of neurodegeneration. Moreover, neuronal death amplifies microglial activation, exacerbating inflammation and oxidative stress, which in turn accelerates disease progression [241].

With respect to astroglia, this cell type plays a key role in the inflammatory processes within the CNS. Astrocytes are crucial in driving chronic neuroinflammation, thereby disturbing homeostasis and increasing the vulnerability of neurons to degeneration and cell death [242]. Astrocytes, the most abundant glial cells in the CNS, contribute to neuroinflammation via the activation of fundamental signaling pathways. Two major transcription factors, NF-κB and AHR, are central players in this regulatory network. NF-κB is a rapidly inducible factor that governs the expression of many pro-inflammatory genes, including cytokines (such as *IL-6* and *TNF-α*), chemokines (such as *CCL2* and *CXCL10*), and adhesion molecules (e.g., *ICAM-1*) [243]. In contrast, AHR has been identified as a potential negative regulator of NF-κB-mediated inflammation, modulating the intensity of inflammatory responses through strong interactions with cytochrome P450 enzymes [244]. The dysregulation of these pathways in astrocytes contributes to a persistent pro-inflammatory state, exacerbating neurodegeneration [245]. Moreover, astrocytes respond to these cytokines, further amplifying inflammatory signaling through JAK/STAT pathways [246].

Oxidative stress is another critical component of age-related neuroinflammation. The accumulation of ROS due to mitochondrial dysfunction leads to the oxidative damage of cellular components, such as lipids, proteins, and DNA [12]. Aged microglia and astrocytes exhibit impaired antioxidant responses, further exacerbating ROS accumulation [247,248]. This oxidative stress not only directly damages neurons but also activates NF-κβ, thereby sustaining the inflammatory cycle. Dysfunctional mitochondria in aging CNS cells exhibit reduced oxidative phosphorylation efficiency, increased mitochondrial DNA mutations, and enhanced cytochrome c release, triggering apoptotic pathways [249].

Aging-related inflammation also affects neurotransmitter systems, disrupting synaptic plasticity and neuronal communication. Pro-inflammatory cytokines modulate neurotransmitter release and receptor expression, leading to excitotoxicity and impaired synaptic function. For example, TNF-α has been shown to disrupt glutamate homeostasis by decreasing the expression of various glutamate transporters (GLT-1 and GLAST) in astrocytes, leading to elevated extracellular glutamate levels and facilitating excitotoxic neuronal damage through excessive NMDA receptor stimulation [250]. Persistent neuroinflammatory and oxidative conditions alter synaptic plasticity by modulating the expression and functionality of key synaptic proteins, including postsynaptic density protein 95 (PSD-95), synaptophysin, and glutamatergic receptor subunits [251]. The breakdown of these proteins impairs long-term potentiation (LTP) and long-term depression (LTD), critical mechanisms underlying learning and memory [252,253].

Moreover, chronic neuroinflammation affects dendritic spine morphology, contributing to the synaptic loss and cognitive decline observed in numerous neurodegenerative diseases [254]. Beyond synaptic dysfunction, the sustained presence of SASP mediators promotes τ hyperphosphorylation, a hallmark of Alzheimer’s disease, through the dysregulation of kinases such as glycogen synthase kinase 3β (GSK-3β) and cyclin-dependent kinase 5 (CDK5). Hyperphosphorylated τ aggregates into insoluble neurofibrillary tangles (NFTs), breaking axonal transport and neuronal communication [255,256]. In Parkinson’s disease, SASP-mediated inflammation enhances α-synuclein misfolding and aggregation via impaired autophagy–lysosomal degradation and proteasomal dysfunction, leading to the accumulation of oligomers that intensify dopaminergic neurodegeneration [257,258]. The persistence of misfolded protein aggregates, coupled with chronic inflammatory and oxidative stress conditions, drives neuronal apoptosis through both caspase-dependent and caspase-independent pathways [259]. Mitochondrial outer membrane permeabilization (MOMP), mediated by the pro-apoptotic Bcl-2 family proteins BAX and BAK, initiates apoptosis by releasing cytochrome c and activating caspase-3 and caspase-9 [260]. Additionally, the excessive activation of PARP-1 in response to DNA damage contributes to parthanatos, a form of programmed cell death distinct from classical apoptosis, further exacerbating neuronal loss [261]. In contrast to classical apoptosis, which is marked by well-defined morphological and biochemical hallmarks such as cell shrinkage, chromatin condensation, and membrane blebbing [262], parthanatos is characterized by a unique series of cellular events, including the overproduction of PAR chains, mitochondrial dysfunction, and the release of apoptosis-inducing factor (AIF) from mitochondria, which subsequently translocates to the nucleus to trigger chromatin condensation and DNA fragmentation [263]. Parthanatos causes irreversible neuronal damage and worsens neuronal loss, particularly in neurodegenerative diseases and acute neuronal injuries, where it interacts with other cell death pathways to accelerate tissue degeneration [264].

## 4. Current Anti-Inflammatory Strategies for Aging

Adopting a healthy lifestyle has long been recognized as one of the most effective strategies for maintaining overall well-being and reducing the impact of aging. A growing body of research supports the idea that some lifestyle factors (e.g., proper nutrition, moderate physical activity, and mental stability) contribute to the mitigation of the aging process. A well-balanced and sufficient nutrient intake has been shown to positively impact aging, with numerous studies suggesting that nutritional factors play an integral role in the modulation of age-related molecular mechanisms. Long-term adherence to a diet rich in polyphenols, naturally occurring compounds in fruits and vegetables, has been particularly associated with significant improvements in intestinal permeability [265,266]. This effect may help maintain the integrity of the intestinal barrier, thereby reducing systemic inflammation [267]. Moreover, polyphenolic compounds have been demonstrated to activate many antioxidant and anti-inflammatory pathways, thus mitigating oxidative stress and promoting cellular resilience [268].

On the other hand, research has revealed that the intake of specific probiotic strains, particularly *Lactobacillus pentosus* var. *plantarum* C29, can reduce SASP factors [269]. The modulation of the gut microbiota through probiotic supplementation results in a reduction in pro-inflammatory cytokine levels and has the potential to decrease the expression of age-associated biomarkers, including tumor suppressor proteins like p16 and p53 [270]. These markers are crucial for cellular senescence and apoptosis, and their regulation has been linked to slowing age-associated cellular dysfunction [271].

Similarly, the intake of polyunsaturated fatty acids (PUFAs), particularly ω-3 fatty acids, has been shown to reduce the levels of pro-inflammatory cytokines [272]. It is thought that reducing inflammation helps preserve metabolic and immune balance, thereby supporting healthier aging [273]. The anti-inflammatory effects of PUFAs extend beyond their role in lipid metabolism and influence gene expression by activating some nuclear receptors such as peroxisome proliferator-activated receptors (PPARs), which regulate lipid homeostasis and inflammatory signaling pathways [274].

Furthermore, the frequent intake of essential vitamins such as vitamin C and vitamin E has been demonstrated to enhance immune cell functions in older adults. Vitamin C, a significant antioxidant, is vital for the proper function of neutrophils, which are critical to immune defense [275]. Specifically, vitamin C facilitates neutrophil chemotaxis and phagocytosis, thereby augmenting the body’s ability to fight infections [276]. Vitamin E, known for its potent antioxidant properties, also plays a fundamental role in stabilizing cellular membranes and protecting immune cells from oxidative damage [277].

Beyond these vitamins, mineral supplementation, particularly zinc, has been identified as a critical factor in supporting immune homeostasis during aging [278]. Zinc is involved in the regulation of a variety of immune processes, including the differentiation and function of T cells [279]. Studies indicate that zinc supplementation can increase the naive T-cell population, which is essential for the body’s ability to respond to new pathogens [280]. Furthermore, zinc contributes to balancing the activity of CD4^+^ T cells, enhancing the immune response and reducing the negative impact of age-related immune dysfunction [281].

Exercise is widely recognized as a highly effective and accessible strategy for mitigating the aging process, functioning through complex biological mechanisms that counteract DNA damage, oxidative stress, and the overall decline in cellular function that often accompanies aging [282]. Recent studies have underscored its role in delaying the aging process at the molecular level, especially through its capacity to regulate telomere length and the activity of crucial enzymes such as telomerase [283]. Research examining middle-aged marathon runners and triathletes has shown that these individuals exhibit significantly higher telomerase activity along with longer telomeres in their circulating white blood cells compared to their sedentary counterparts. This finding suggests that endurance exercise not only helps maintain telomere integrity but may also promote the rejuvenation of immune cells, enhancing overall immune function and mitigating age-related declines in immune response [284]. This evidence indicates that endurance exercise helps preserve telomere integrity and rejuvenates immune cells, improving immune function and delaying age-related immune decline. The underlying mechanisms likely involve reducing oxidative stress, modulating inflammation, and activating genes that promote cell survival and repair [285,286]. In contrast, resistance training offers benefits for combatting aging at the cellular level, with studies showing significant improvements in the health of adipose tissue in older and obese patients [287]. It was discovered that resistance exercise led to a reduction in the number of cells expressing p16, a protein marker commonly associated with cellular senescence [288]. By reducing the number of p16-expressing cells in thigh adipose tissue, resistance training appears to foster a more youthful and functional tissue environment, potentially by promoting the turnover of damaged cells and alleviating inflammation in adipose tissue [289].

Emerging research has increasingly highlighted the pro-inflammatory cytokine network as a crucial target for anti-aging interventions, focusing on the potential to alleviate chronic inflammation, which is one of the key drivers of age-related diseases and cellular dysfunction [290]. In light of this, various anti-inflammatory compounds have garnered significant attention for their ability to counteract inflammation, promote cellular health, and potentially delay the onset of age-related decline. Among these, metformin, a widely used medication for managing type 2 diabetes, has emerged as a promising candidate for promoting healthy aging [291]. Metformin has been shown to influence the IKK/NF-κB pathway, a primary regulator of inflammation, as well as the GPX7/NRF2 axis, which is essential for cellular defense against oxidative stress and the regulation of antioxidant responses [292,293]. Recent research has revealed that metformin interacts with PEN2, an identified target involved in age-related inflammation [294]. By modulating all of these pathways, metformin reduces inflammation and promotes healthier aging, potentially extending lifespan [291]. Similarly, aspirin reduces oxidative damage, maintains tissue function, promotes cellular regeneration, and eliminates SASP mediators, thereby presenting a potential therapeutic strategy for age-related tissue degeneration [295].

Finally, among the broad spectrum of cellular anti-aging therapies, senolytics (agents designed to selectively eliminate senescent cells) emerge as one of the most promising yet highly debated strategies in gerontology [296]. Since their discovery in 2015 [297], several compounds have moved from initial discovery to clinical trials, resulting in considerable enthusiasm within the scientific community. Senolytic drugs can be classified into phytochemicals and proprietary compounds, differing in their origin, composition, regulatory oversight, and mechanisms of action. Phytochemicals are naturally occurring compounds derived from plants, often associated with potential health benefits and therapeutic properties. They are found in several foods, herbal medicines, and dietary supplements, with their biological effects typically attributed to the synergistic interactions of multiple bioactive constituents [297,298,299]. In contrast, proprietary drugs are synthetic compounds developed by pharmaceutical companies. These drugs undergo thorough testing, including clinical trials, to assess their safety, efficacy, and standardized dosing before gaining regulatory approval [299,300].

Among the most studied senolytic agents are dasatinib and quercetin. Dasatinib has shown effectiveness in eliminating senescent human adipocyte progenitor cells, which reside in adipose tissue and contribute to age-related metabolic dysfunction [301,302]. Quercetin has proven potent in clearing senescent endothelial cells, which line the blood vessels, as well as bone marrow stem cells in murine models [303]. These two drugs constitute a combination therapy that has shown significant efficacy in targeting various types of senescent cells, thereby reducing inflammation, enhancing tissue function, and alleviating the burden of age-related diseases in animal models [301,304,305,306]. Fenofibrate, primarily used for cholesterol management, has emerged as a promising therapeutic candidate due to its ability to clear senescent cells by upregulating PPARα [307]. Ultimately, taurine and spermidine have emerged as promising senolytic agents in the context of aging, demonstrating potential in promoting cellular homeostasis and mitigating age-related decline. Taurine, a conditionally essential amino acid, has been shown to enhance mitochondrial function, reduce oxidative stress, and modulate inflammatory responses, thereby contributing to the clearance of senescent cells [308]. Similarly, spermidine, a polyamine present in youthful cells, has been strongly implicated in autophagy induction, chromatin stabilization, and the suppression of pro-inflammatory pathways, all of which are fundamental for maintaining cellular integrity and delaying age-associated pathologies [309].

## 5. Conclusions

In conclusion, this review presents a thorough analysis of the complex biological processes that underpin aging and its connection to various pathological conditions. Aging encompasses several intricate physiological changes that significantly impact the body’s ability to maintain homeostasis. This loss of homeostasis plays a role in the development of chronic diseases commonly associated with aging, such as cardiovascular disease and neurodegenerative disorders, by disrupting cellular activities, impairing the body’s ability to repair tissues, and increasing its vulnerability to inflammation and oxidative stress.

One of the key themes explored in this article is the role of inflammation in the aging process. Chronic low-grade inflammation, also known as inflammaging, is highlighted as a hallmark of aging that drives the progression of these age-related diseases. Unlike the acute, short-lived inflammation that arises from injury or infection, inflammaging is a sustained, low-grade inflammation that can inflict lasting damage on the body. This chronic inflammatory state accelerates the decline of cellular and tissue function by inducing tissue damage and impairing the body’s capacity for self-repair.

Furthermore, this article explores the connection between aging and immune system dysfunction, which plays a key role in the initiation and progression of diseases in older adults. Immunosenescence, the progressive deterioration of immune function, impairs the body’s ability to protect against pathogens and injuries. As the immune system weakens, older individuals become more susceptible to infections, autoimmune disorders, and cancer. These processes not only induce inflammation but also result in the accumulation of dysfunctional cells, thereby exacerbating the pathological effects of aging.

This research highlights the importance of identifying therapeutic strategies to mitigate the effects of aging and chronic inflammation. Targeting specific inflammatory mediators and promoting immune rejuvenation could play a fundamental role in extending healthspan (the period of life spent in good health). Furthermore, delaying cellular senescence, the process by which cells lose their ability to divide and function, could help maintain tissue integrity and enhance overall health in aging populations.

Finally, this review calls for a more cohesive healthcare approach for the aging population, one that addresses not only the symptoms of age-related diseases but also the root causes of aging itself. By focusing on the fundamental mechanisms of aging and chronic inflammation and implementing personalized interventions, it is possible to encourage healthier aging, enhance quality of life, and increase the resilience of older adults.

## Data Availability

Not applicable.

## Abbreviations

The following abbreviations are used in this manuscript:

4-HNE	4-hydroxy-2-nonenal
ACTH	Adrenocorticotropic hormone
AGE	Advanced glycation end-product
AHR	Aryl hydrocarbon receptor
AIF	Apoptosis-inducing factor
AKT/PKB	Protein kinase B
AMPA	Alpha-amino-3-hydroxy-5-methyl-4-isoxazolepropionic acid
ATP	Adenosine triphosphate
BAK	Bcl-2 homologous antagonist/killer
BAX	Bcl-2-associated X protein
BBB	Blood–brain barrier
Bcl-2	B-cell lymphoma 2
BPH	Benign prostatic hyperplasia
CCL2	C-C motif chemokine ligand 2
CCL4	C-C motif chemokine ligand 4
CD25	Cluster of differentiation 25
CD4	Cluster of differentiation 4
CDK4/6	Cyclin-dependent kinases 4 and 6
CDK5	Cyclin-dependent kinase 5
CMA	Chaperone-mediated autophagy
CNS	Central nervous system
COPD	Chronic obstructive pulmonary disease
COX-1	Cyclooxygenase 1
COX-2	Cyclooxygenase 2
CRH	Corticotropin-releasing hormone
CXCL10	C-X-C motif chemokine ligand 10
CXCL12	C-X-C motif chemokine ligand 12
CXCR4	C-X-C chemokine receptor type 4
CYP3A4	Cytochrome P450 3A4
DAMPs	Damage-associated molecular patterns
DC	Dendritic cell
DDR	DNA damage response
DHEA	Dehydroepiandrosterone
DNA	Deoxyribonucleic acid
DPPC	Dipalmitoylphosphatidylcholine
ECM	Extracellular matrix
EGFR	Epidermal growth factor receptor
eNOS	Endothelial nitric oxide synthase
ERK	Extracellular signal-regulated kinase 1/2
FoxP3	Forkhead box P3
GDF-15	Growth differentiation factor 15
GERD	Gastroesophageal reflux disease
GH	Growth hormone
GHRH	Growth hormone-releasing hormone
GI	Gastrointestinal system
GLAST	Glutamate aspartate transporter
GLT-1	Glutamate transporter 1
GM-CSF	Granulocyte–macrophage colony-stimulating factor
GnRH	Gonadotropin-releasing hormone
GPX7	Glutathione peroxidase 7
GSK-3β	Glycogen synthase kinase 3 beta
HSP70	70 kDa heat shock protein
ICAM-1	Intercellular adhesion molecule 1
IFN-γ	Interferon gamma
IGF-1	Insulin-like growth factor 1
IGFBP-2	Insulin-like growth factor binding protein 2
IKK	IκB Kinase
IL-10	Interleukin 10
IL-12	Interleukin 12
IL-17	Interleukin 17
IL-18	Interleukin 18
IL-1α	Interleukin 1 alpha
IL-1β	Interleukin 1 beta
IL-2	Interleukin 2
IL-4	Interleukin 4
IL-6	Interleukin 6
IL-8	Interleukin 8
iNOS	Inducible nitric oxide synthase
LPS	Lipopolysaccharide
LTD	Long-term depression
LTP	Long-term potentiation
MAPK	Mitogen-activated protein kinase
MDA	Malondialdehyde
MEK	Mitogen-activated protein kinase kinase
MMP	Matrix metalloproteinase
MMP-13	Matrix metalloproteinase 13
MMP-2	Matrix metalloproteinase 2
MMP-3	Matrix metalloproteinase 3
MMP-9	Matrix metalloproteinase 9
MOMP	Mitochondrial outer membrane permeabilization
NADPH	Nicotinamide adenine dinucleotide phosphate (reduced form)
NFT	Neurofibrillary tangle
NF-κB	Nuclear factor kappa-light-chain-enhancer of activated B cells
NLRP3	NLR family pyrin domain containing 3
NMDA	N-methyl-D-aspartate
NRF2	Nuclear factor erythroid 2-related factor 2
NSAID	Non-steroidal anti-inflammatory drug
p16	Cyclin-dependent kinase inhibitor 2A
p21	Cyclin-dependent kinase inhibitor 1
P450	Cytochrome P450
p53	Tumor protein P53
PAR	Poly (ADP-ribose)
PARP-1	Poly (ADP-ribose) polymerase 1
PEN2	Presenilin enhancer 2
PI3K	Phosphoinositide 3-kinase
PPAR	Peroxisome proliferator-activated receptor
PPARα	Peroxisome proliferator-activated receptor alpha
PSD-95	Postsynaptic density protein 95
PTEN	Phosphatase and tensin homolog
PTM	Post-translational modification
PUFA	Polyunsaturated fatty acid
RAAS	Renin–angiotensin–aldosterone system
Rb	Retinoblastoma
RNA	Ribonucleic acid
RNS	Reactive nitrogen species
ROS	Reactive oxygen species
Runx2	Runt-related transcription factor 2
SASP	Senescence-associated secretory phenotype
SCFA	Short-chain fatty acid
T3	Triiodothyronine
T4	Thyroxine
TGF-β	Transforming growth factor beta
TIMP	Tissue inhibitor of metalloproteinase
TLR	Toll-like receptor
TLR4	Toll-like receptor 4
TNFR	Tumor necrosis factor receptor
TNF-α	Tumor necrosis factor alpha
TP53	Tumor protein p53
Treg	Regulatory T cell
UPS	Ubiquitin–proteasome system
VCAM-1	Vascular cell adhesion molecule 1
VEGF	Vascular endothelial growth factor
ZO	Zonula occludens
